# Risk factors for the development of reexpansion pulmonary edema in patients with spontaneous pneumothorax

**DOI:** 10.1186/1749-8090-8-164

**Published:** 2013-07-01

**Authors:** Jeong-Seob Yoon, Jong-Hui Suh, Si Young Choi, Jong Bum Kwon, Bae Young Lee, Sang Haak Lee, Chi Kyung Kim, Chan Beom Park

**Affiliations:** 1 Department of Thoracic and Cardiovascular Surgery, Incheon St. Mary’s Hospital, The Catholic University of Korea, Gyeonggi-do, South Korea; 2 Department of Thoracic and Cardiovascular Surgery, Uijeongbu St. Mary’s Hospital, The Catholic University of Korea, Gyeonggi-do, South Korea; 3 Department of Thoracic and Cardiovascular Surgery, Daejeon St. Mary’s Hospital, The Catholic University of Korea, Seoul, Republic of Korea; 4 Department of Radiology, St. Paul’s Hospital, The Catholic University of Korea, Gyeonggi-do, South Korea; 5 Departments of Internal Medicine, Division of Pulmonology, St. Paul’s Hospital, The Catholic University of Korea, Gyeonggi-do, South Korea; 6 Department of Thoracic and Cardiovascular Surgery, St. Paul’s Hospital, The Catholic University of Korea, Gyeonggi-do, South Korea

**Keywords:** Pneumothorax, Reexpansion Pulmonary Edema, Diabetes Mellitus

## Abstract

**Background:**

Reexpansion pulmonary edema (REPE) is known as a rare and fatal complication after tube thoracostomy.

**Objectives:**

We investigated the risk factors for the development of REPE in patients with spontaneous pneumothorax.

**Methods:**

We selected patients who were diagnosed with spontaneous pneumothorax and were initially treated with tube thoracostomy between August 1, 2003 and December 31, 2011. The patients’ electronic medical records, including operative notes and chest x-ray and computed tomography scans, were reviewed.

**Results:**

REPE developed in 49 of the 306 patients (16.0%). REPE was more common in patients with diabetes (14.3% vs 3.9%, *P* = 0.004) or tension pneumothorax (46.8% vs 16.2%, *P* = 0.000). The pneumothorax was larger in patients with REPE than without REPE (57.0 ± 16.0% vs 34.2 ± 17.6%, *P* = 0.000), and the incidence of REPE increased with the size of pneumothorax. On multivariate analysis, diabetes mellitus [(odds ratio (OR) = 9.93, *P* = 0.003), and the size of pneumothorax (OR = 1.07, *P* = 0.000) were independent risk factors of REPE.

**Conclusions:**

The presence of diabetes increases the risk of REPE development in patients with spontaneous pneumothorax. The risk of REPE also increases significantly with the size of pneumothorax.

## Background

Reexpansion pulmonary edema (REPE) is generally known as a rare but potentially fatal complication after treatment of pneumothorax, hemothorax and pleural effusion [1,2]. Several factors have been implicated in the pathogenesis of REPE: rapid reexpansion, application of negative intrapleural pressure, decreased surfactant activity, increased pulmonary vascular permeability, airway obstruction, pulmonary artery pressure change, and chronicity of lung collapse [3,4]. Recent studies support increased pulmonary capillary permeability as an important etiologic factor [3-6]; however, the pathogenesis of REPE remains unclear. The objectives of this study were to determine the risk factors for the development of REPE after tube thoracostomy in patients with spontaneous pneumothorax.

## Methods

The institutional review board of St. Paul’s hospital approved this study, with a waiver of the informed consent (no. PC12RISI0058).

We identified 397 episodes in 321 patients who were diagnosed with spontaneous pneumothorax between August 1, 2003 and December 31, 2011. The patients’ electronic medical records, including operative notes and chest x-ray and computed tomography scans, were retrospectively reviewed.

After excluding 23 episodes that were treated by oxygen supplementation only, 67 episodes that were operated without tube thoracostomy, and 1 patient who was transferred from another hospital with a tube thoracostomy, 306 episodes of spontaneous pneumothorax that were initially treated with closed tube thoracostomy drainage were included in the study population. Negative pressure suction drainage was not routinely connected. The patients’ electronic medical records and chest radiographs and chest computed tomography (CT) scans were analyzed. The Chest CT scan was checked in 257 (84.0%) patients, and only cases with a history of chest tube insertion within 24 hours were included in the study.

Chest x-RAY and CT scans that were obtained in the same center were reviewed by a thoracic radiologist (B.Y.L.) and a thoracic surgeon (C.B.P.) without the clinical information of the patients. REPE was defined radiologically as the presence of the following features [7,8]: (1) ipsilateral ground-glass opacities, (2) interlobular septal thickening or intralobular interstitial thickening, (3) consolidation, and (4) atelectasis. Other causes for these findings such as malignancy, pneumonia, and obstructive pneumonopathy were excluded.

The area of pneumothorax (Area_pneumothorax_) and involved hemithorax (Area_hemithorax_) was measured on a chest radiograph using a picture archiving communication system (PACS; Marosis m-view, Infinitt, Korea) with an automated region of interest (ROI) calculator by a thoracic radiologist (B.Y.L.) and a thoracic surgeon (C.B.P.). The pneumothorax size was determined using the following formula:

$$\begin{array}{c} \mathrm{Size}\;\mathrm{of}\;\mathrm{pneumothorax}\;\left(\%\right)\\ \;=\left({\mathrm{Area}}_{\mathrm{pneumothorax}}/{\mathrm{Area}}_{\mathrm{hemithorax}}\right)\times \;100 \end{array}$$

**Figure 1:**
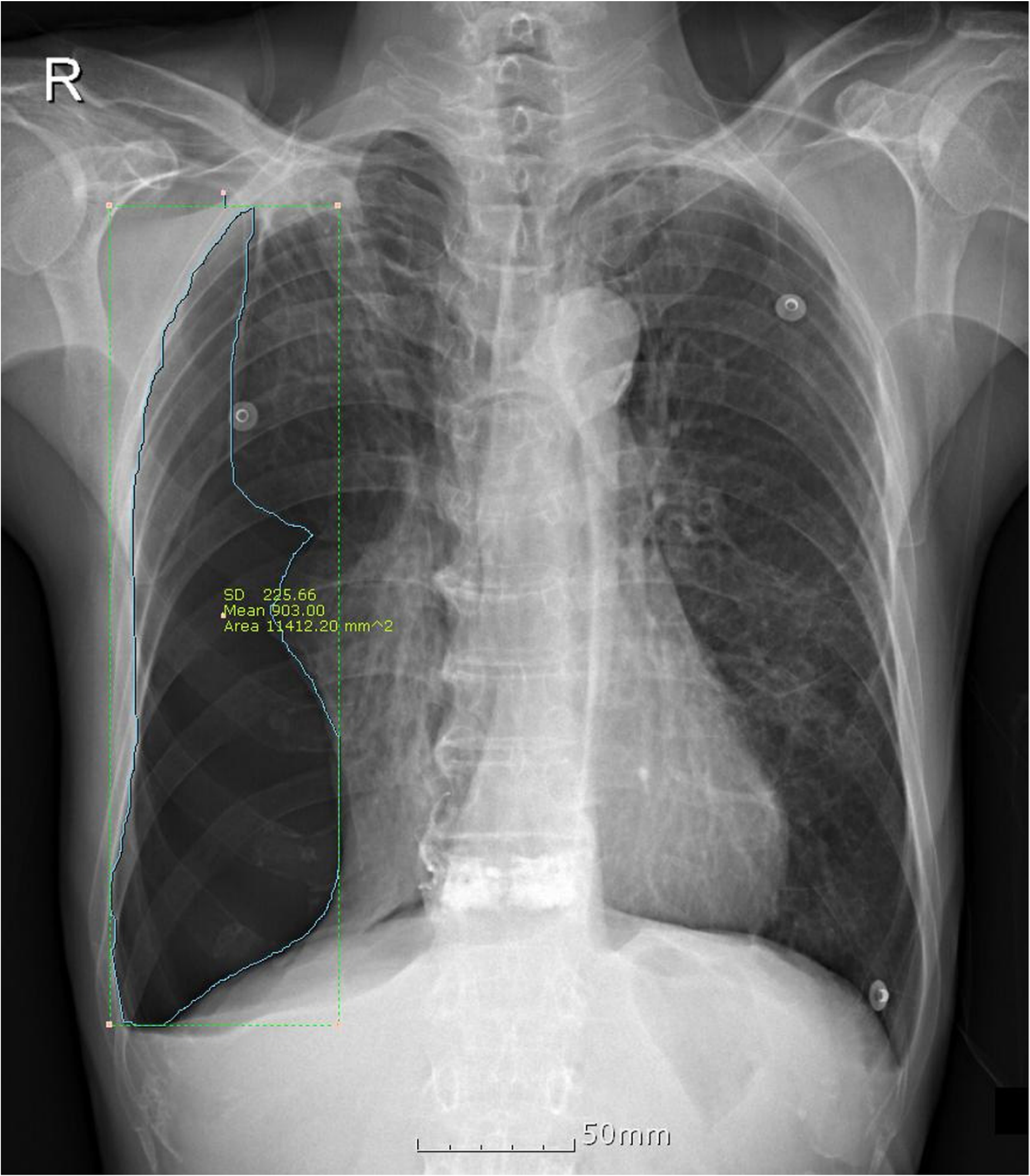
**Measuring the size of pneumothorax.** The area of pneumothorax were measured on a chest radiograph using an automated region of interest caculator in a picture archiving communication system.

Rhea’s method [9] is a simple and popular way of measuring the size of pneumothorax based on the average interpleural distance. Their formula is as follows; Pneumothorax size (%) = Average interpleural distance (cm) = (maximum apical interpleural distance + interpleural distance at the midpoint of upper half of the lung + interpleural distance at the midpoint of the lower half of the lung)/3. However, it has the limitation of measuring the extent of pneumothorax in the presence of fibrotic adhesion [10]. The PACS automated ROI calculator can calculate the pneumothorax size irrespective of the shape of the collapsed lung (Figure 1).

In the following, “total events” refers to the sum of the frequency of ipsilateral and contralateral occurrences of pneumothorax, “ipsilateral recurrence” indicates the number of occurrences of an ipsilateral pneumothorax, and “fibrotic adhesion” is defined as the radiologic findings of diffuse joining of the parietal pleura and the visceral pleura or the presence of adhesive band.

### Statistical analysis

Continuous variables are presented as mean ± standard deviation values, and categorical variables are summarized as frequencies and percentages. Continuous variables were compared using the independent two-sample *t* test or Mann–Whitney *U* test, and categorical variables were compared using the chi square or Fisher’s exact test, as appropriate. The variables included in the multivariate analysis were those detected by univariate models as having a significant association (*P* < 0.05). Independent risk factors for REPE were determined using stepwise logistic regression analysis. The statistical analyses were performed using SPSS version 17.0 (SPSS, Chicago, Illinois, USA).

## Results

Overall, of the 306 patients with tube thoracostomy, REPE developed in 49 patients (16.0%). The characteristics of the study population are listed in Table 1. The REPE and no-REPE groups were demographically similar. The age of all patients was 44.1 ± 22.1 years, with men being predominant gender (85.6% vs 14.4%). Primary pneumothorax was 53.3% and secondary pneumothorax 46.7%. The incidence of a first-time episode was 74.2%, and that of a recurrent event was 25.8%. The pneumothorax was the right side in 52.6% and the left side in 47.4%. Tension pneumothorax occurred in 60 patients (19.6%) and fibrotic adhesion was found in 110 patients (35.9%) (Table 2).

**Table 1:** Patient characteristics

**Variable**	**With reexpansion pulmonary edema (*n*** **= 49)**	**Without reexpansion pulmonary edema (*n*** **= 257)**	***P *value**
Age (years)	46.7 ± 20.1	43.7 ± 22.5	0.253
Sex (male)	39 (79.6%)	223 (86.8%)	0.189
Diagnosis			0.690
Primary	25 (51.0%)	138 (54.1%)	
Secondary	24 (49.0%)	117 (45.9%)	
Diabetes mellitus	7 (14.3%)	10 (3.9%)	0.004
Hypertension	8 (16.3%)	31 (12.1%)	0.412
Tuberculosis	17 (34.7%)	81 (31.5%)	0.662
COPD	15 (30.6%)	78 (30.4%)	0.971
Smoking	27 (57.4%)	108 (46.2%)	0.157
Total events	1.20 ± 0.65	1.46 ± 0.90	0.034
Ipsilateral recurrence	1.12 ± 0.33	1.30 ± 0.62	0.090
Site (right : left)	28 : 21	132 : 125	0.458
Bilateral pneumothorax	2 (4.1%)	3 (1.2%)	0.183
Serum albumin (gm/dl)	4.18 ± 0.48	4.27 ± 0.48	0.226

*COPD*, Chronic obstructive pulmonary disease.

**Table 2:** Radiologic findings

**Variable**	**With reexpansion pulmonary edema (*n*** **= 49)**	**Without reexpansion pulmonary edema (*n*** **= 257)**	***P *value**
Tension pneumothorax	22 (46.8%)	38 (16.2%)	0.000
Fibrotic adhesion	17 (36.2%)	93 (39.7%)	0.647
Size of pneumothorax (%)	57.0 ± 16.0	34.2 ± 17.6	0.000
Size of largest bullae (mm)	18.7 ± 18.6	19.8 ± 20.6	0.949
Number of bullae			0.840
0	4 (8.9%)	18 (8.5%)	
1	6 (13.3%)	35 (16.5%)	
2	5 (11.1%)	22 (10.4%)	
3	3 (6.7%)	26 (12.3%)	
4	4 (8.9%)	12 (5.7%)	
≥5	23 (51.1%)	99 (46.7%)	

**Figure 2:**
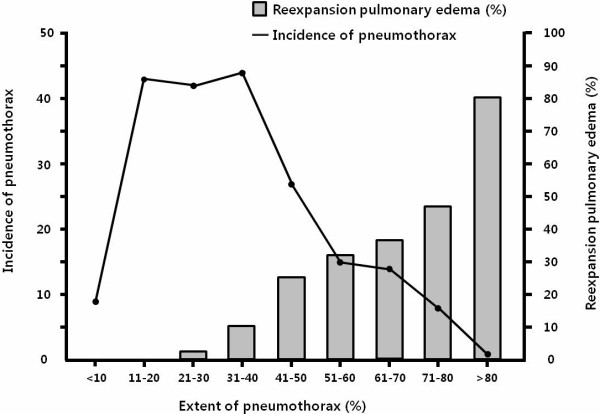
**The incidence of reexpansion pulmonary edema (REPE) increased with the size of pneumothorax.** Gray rectangle, proportion of REPE; Black line, incidence of pneumothorax.

The extent of pneumothorax was greater with REPE than without REPE (57.0 ± 16.0% vs 34.2 ± 17.6%, *P* = 0.000) (Table 1), and the incidence of REPE increased with the size of pneumothorax (Figure 2). Diabetes mellitus was more common among REPE patients than among those without REPE (14.3% vs 3.9%, *P* = 0.004). The size and the number of bullae did not differ significantly between the groups. The level of serum albumin also did not differ between those with and those without REPE (4.18 ± 0.48 vs 4.27 ± 0.48, *P* = 0.226).

The independent risk factors for the development of REPE were identified by multivariate analysis as diabetes mellitus [odds ratio (OR) = 9.93, 95% confidence interval (CI) = 2.17-45.49, *P* = 0.003)], and a 10% increase in the size of pneumothorax (OR = 1.07; 95% CI = 1.04-1.09, *P* = 0.000)(Table 3).

**Table 3:** Multivariate analysis for the development of reexpansion pulmonary edema

**Variable**	**Odds ratio**	**95% Confidence interval**	***P *value**
Total events	0.78	0.39 – 1.58	0.494
Diabetes mellitus	9.93	2.17 – 45.49	0.003
Tension pneumothorax	2.19	0.95 – 5.05	0.067
Size of pneumothorax^a^	1.07	1.04 – 1.09	0.000

^a^ 10% size increase.

## Discussion

The findings of the present study demonstrate that diabetes is an important risk factor of REPE in patients with spontaneous pneumothorax. To our knowledge, this is the first time that diabetes has been shown to contribute to the development of REPE.

Diabetes mellitus causes vascular, renal, retinal, and neuropathic complications. While the mechanisms underlying the diabetic degenerative complications are still not completely understood, microangiopathy is an important pathophysiologic mechanism, initially it causing damage to the basement membrane; basement membrane thickening is the histological hallmark of diabetic microangiopathy. Microangiopathy occurs commonly during the course of diabetes, leading to damage not only to the kidneys, eyes, and nervous system, but also to the pulmonary alveolar basement membrane [11,12]. These histological findings were demonstrated in the experimental evaluation of REPE [13].

The association between pneumothorax and diabetes mellitus is not known. Thickening of the pulmonary alveolar basement membrane has been shown in types 1 and 2 diabetes mellitus in autopsy studies [13], and Vracko et al. [12] reported that diabetes leads to thickening of the alveolar epithelial and capillary basal lamina. Recent studies have demonstrated a relationship between basement membrane thickening and increased vascular permeability in the high-glucose condition [14,15]. Thickening of the basement membrane in the high-glucose condition is related to increased fibronectin and collagen IV protein levels [14] and decreased levels of heparan sulfate proteoglycan, which restrict the passage of protein across the basement membrane [16]. These structural and biochemical changes in the basement membrane allows increased permeability [14].

Several authors have suggested that increased pulmonary capillary permeability is a major factor in the development of REPE [3-6,13,17]. The cause of the increased capillary permeability is unclear. The thickened basement membrane and alterations of the composition of extracellular matrix in diabetic patients could be the cause of pulmonary edema during reexpansion.

The lung extracellular matrix contributes to the mechanical tensile and compressive strength, elasticity, and the maintenance of normal interstitial fluid dynamics [18]. Chronic lung collapse thickens the pulmonary capillary endothelium and the basement membrane [13]. Physical stimuli on endothelial cell surface lead to biochemical and biophysical changes in the plasma membranes and increase the tissue forces at interstitial level, thus increasing the thickness of the extracellular matrix. In pulmonary edema, changes in the levels of heparan sulfate proteoglycan augmented the microvascular permeability [16,19] and increase the level of metalloproteinases [20].

Exposure of the pulmonary vasculature to mechanical stress during reexpansion may contribute to the increased vascular permeability. Mechanical stress associated with lung collapse affects the endothelium and changes the pulmonary lymphatic flow, and stretching during reexpansion of the lung injuries the pulmonary microvessels. This injury reduces alveolar surfactant activity [13] and the intrapleural and perivascular pressure. Oxygen-derived free radicals are produced in the injured microvessels during reexpansion [21], and migrated leukocytes exacerbate the damage to the pulmonary microvessels, producing pulmonary edema [22].

Several studies have demonstrated that the incidence of REPE is higher when the size of the pneumothorax is larger [10,23,24]. These finding are consistent with our results. We also found that the development of REPE was correlated with the increase of the size of pneumothorax (Figure 2). A large pneumothorax induces more thickening and hardening of the pulmonary microvascular endothelium. During reexpansion, the increased tensile stress leads to more injury in the pulmonary microvessels, and the consequent structural and biochemical changes could result in pulmonary edema. Matsuura et al. [23] indicated that the severity of collapse of the lung is a major determining factor in promoting REPE, and showed that aging protects against the occurrence of REPE in the older subjects (the incidence of REPE was lower in their patients aged >40 years than in those aged 20-39 years). Unlike the study of Matsuura et al., we did not find any relationship between age and the incidence of REPE; furthermore, old age did not appear to be protective. One possible explanation for their result is that pneumothorax was more severe in their patients aged 20-39 years than in those aged >40 years.

Our study has limitations. First, we used the PACS automated ROI calculator system; however, pneumothorax is three-dimensional rather than two-dimensional, and three-dimensional measurement of pneumothorax using CT data is more accurate than two-dimensional measurement using chest x-ray. However, pneumothorax size as measured on a chest radiograph is correlated with the CT-based volume measurement [9,25]. Our treatment policy is perform CT after tube thoracostomy, and so three-dimensional measurement of the pneumothorax volume using CT might be available in a prospective study. Our study included 110 cases (35.9%) of fibrotic adhesion. In cases of fibrotic adhesion the method of Rhea et al. [9] and Collins et al. [25] using the interpleural distance is not available. Our method could enable more accurate measurement of the size of the pneumothorax even when it has an irregular shape. Second, the radiologic definition of REPE was applied in this study. Radiologically defined REPE is more common than clinically defined REPE; however, it is reasonable to assume that the pathogenesis of these two conditions is similar. Third, the wide confidence interval (2.17 – 45.49) is associated with the relatively small number of diabetes in this study. However, the probability value of 0.003 indicated that diabetes is statistically significant.

## Conclusions

In conclusion, REPE is not uncommon and fatal outcome is extremely rarer than previously reported. Diabetes is an independent risk factor for the development of REPE in patients with spontaneous pneumothorax. The risk of REPE also increases significantly with the size of pneumothorax.

## Abbreviations

REPE: Reexpansion pulmonary edema; CT: Computed tomography; ROI: Region of interest; PACS: Picture archiving communication system; OR: Odds ratio; CI: Confidence interval.

## Competing interests

The authors declare that they have no competing interests.

## Authors’ contribution

JS Yoon contributed to writing and critical revision of the manuscript. JH Suh contributed to statistical analysis. SY Choi contributed to writing the manuscript. JB Kwon contributed to collecting data. BY Lee contributed to analysis of radiologic findings. SH Lee contributed to statistical analysis. CK Kim contributed to interpretation of data. CB Park was responsible for the integrity of the work and edited manuscript. All authors read and approved the final manuscript.
